# Chloroform

**Published:** 1861-07

**Authors:** 


Chloroform.—*A Suggestion as to the Cause of Fatal Results from its Inhalation.*—There has been much discussion of late upon the fatality caused by the inhalation of chloroform. We know several surgeons who will not use it under any circumstances. Within the last year we have met with three peculiar cases in which the patient seemed upon the point of suffocation. In the first of these a large spoon was near, which was seized and thrust into the mouth, and two fingers thrust over it to the throat. The depression and bringing forward of the tongue by this means caused an instantaneous gasp, and breathing was resumed. The other two cases were similar, the tongue being thrown back, closing the epiglotis ; relief was given in the manner before described. While in Albany, a short time since, these cases were mentioned to Dr. Swinburne of that city. He said that not only had he arrived at the same conclusion, that death from inhalation of chloroform was in most instances caused by suffocation, owing to the tongue falling back and preventing air from entering the lungs, but that he had performed experiments upon dogs, *and demonstrated it to be a fact.* To one dog he had given one and three-quarter pounds of chloroform, and several times during its inhalation, respiration ceased, but was recommenced by drawing the tongue forward. He said that respirations did not cease if steadily administered for a length of time, when the tongue was kept well forward. It is only necessary to mention the fact ; if a discussion were necessary upon the subject, we can give our theory and support it by the result of the published autopsies.—*Communicated to American Medical Times, by Dr. Saml. R. Percy.*

Indian Hemp.—The *Archives of Medicine* No. vii. contains an article from the pen of Dr. J. Russell Reynolds of London, "On some of the Therapeutical Uses of Indian Hemp," in which the author claims for the drug a good share of confidence, when a proper case is selected, a pure drug employed, and a proper dose exhibited; and that the uncertainty of its action is due to the failure of one of these three conditions. He says, "Hemp is a soporific, anodyne, and anti-spasmodic —it relieves pain, and spasm, and conduces to sleep; in doing either of these, it usually promotes diaphoresis and diuresis; whereas it does not leave behind it headache or vertigo; nor does it affect the appetite nor confine the bowels.

Its beneficial effects are illustrated, 1. In cases of mental or emotional disturbance.—A remarkably intelligent boy, æt. 8, complained for four or five months, of frequent headaches, troublesome dreams, uneasy sleep with sighing respiration, etc. The sixth of a grain, taken every evening, soon restored perfect tranquillity. A merchant, who had suffered from yellow fever, became "excessively depressed in spirits, haunted with the gloomiest apprehensions and suicidal thoughts," nights restless. Extr. cannab. Ind. gr. ss., o. n., soon insured him good nights and days. A gentleman, æt. 78, mental powers failing, had been threatened with paralysis, and became extremely restless at night. A dose of gr. $\frac{1}{3}$ to gr. $\frac{2}{3}$ would induce sleep within ten minutes. This was continued for many months with the same success, it never being necessary to increase the dose. 2. For the relief of certain kinds of pain:—A young gentleman, who had suffered for several years from intense pain in the jaws, face, and head, was relieved by gr. $\frac{1}{3}$ can. Ind., forma pil., o. n. Tr. ferri sesquichlor. ʒ ss., t.d. An intelligent boy, æt. 7, was first noticed to clench his left hand involuntarily, afterwards suffering from violent headaches, located in the forehead, occurring once a week, followed by partial paralysis of the left side. Was relieved by can. Ind. gr. $\frac{1}{4}$, bis die, with potass. iod. gr. iv., and dec. cinch. ℥ j. A gentleman, æt. 59, suffered for twenty years from pain in right scapula, and corresponding portion of the spine; afterwards numbness and tingling down the arm similar to that produced by pressing on the nerve at the elbow. Extr. can. Ind. gr. $\frac{1}{3}$, t.d., forma pilulæ sin. camph. e. opio, pro usu; syr. ferri iodidi, ♏xxx, t.d. "Within a fortnight the pain was completely removed; the tingling sensation, however, persisted." A clergyman, æt. 70, complained of pain in left side of neck and back, extending to the head, followed by difficulty in articulation. Tongue deviated to the left, head drawn towards left shoulder, arcus senilis marked, spirits depressed. The pain was relieved by can. Ind. gr. $\frac{1}{3}$, ter die. A young lady of highly nervous temperament was relieved of severe attacks of hemorrhage by gr. $\frac{1}{3}$ doses given thrice daily. 3. In certain forms of convulsions :—An officer in a cavalry regiment, æt. 28, had suffered from slight epileptic attacks, gradually increasing in severity, until they at length became frequent and tetaniform. Though not entirely cured, the severity of the fits was greatly relieved by gr. $\frac{1}{3}$ doses every three hours. A gentleman, æt. 45, of good general health, but subject to frequent excitement, was suddenly seized with a violent convulsion, followed by heavy and stertorous sleep, and after by maniacal excitement for fifteen minutes, which passed into another fit, passing through a similar series of symptoms about once an hour. After failure of the ordinary methods of treatment, gr. i. of can. Ind. was given, and rejected by the stomach. Another dose was given and retained, which afforded complete relief. By the same treatment, a case of obstinate vomiting, in a young lady, was entirely cured, and an epileptic youth was greatly relieved. On the other hand, it was absolutely useless in most cases of epilepsy, hypochondria, and the various hysterical affections. To give a bird's eye view of the whole subject, the remedy was for the *relief of emotional disturbances.*

| SUCCESSFUL IN | UNSUCCESSFUL IN |
|---|---|
| 1. Deranged cerebral circulation, with pain and delirium. | 1. Hypochondriasis. |
| 2. Incipient insanity after yellow fever. | 2. Temporary, recurrent religious melancholy. |
| 3. Senile remollissement. | 3. Insomnia with diabetes. |

### *Painful Affections.*

| | |
|---|---|
| 1. Nervous irritation from carious teeth. | 1. Sciatica. |
| 2. Probable tumor of brain. | 2. Hysterical hip-joint. |
| 3. Probable thickening of spinal meninges. | 3. Hysterical headache. |
| 4. Hemorrhage at roots of 8th and 9th nerves. | |
| 5. Syphilitic meningitis. | |
| 6. Hemiceanir. | |

*Affections of Motility.*

| | |
|---|---|
| 1. Meningitis. | 1. Epilepsy. |
| 2. Intense cerebral congestion. | |
| 3. Obstinate nervous vomiting. | |
| 4. Recurrent convulsions. | |

It does not, like opium, purchase present relief at the expense of future misery. The value of the medicine seems enhanced, because the limitation of its action will enable us to apply it with scientific selection.—*Am. Medical Times.*

---

WATER: ITS HISTORY, CHARACTERISTICS, HYGIENIC, AND THERAPEUTIC USES.—By *Samuel W. Francis, A. M., M. D.* —No. 1.—*Religious History.*—When the pleasing duty is assigned us of discussing a subject which could well employ the thoughtful mind for many busy years, we enter upon a consideration of its beauty and depths with a calm pensiveness, and pursue its charms with a quiet enjoyment, unlimited by the narrow bounds of a set task. We feel that humanity is interested in the same object of our investigation, and appreciate, to the fullest extent, the contemplation of that which is common, in its usefulness, to all, and may, with impunity, be regarded by mankind as the only true democratic link in nature.

Water, as a theme, is as expansive as the ocean—as endless, in its teachings of philosophy, as the globe itself—and as truthful in its reflections as it is pure in its organization: as an inhabitant of the earth, one of the oldest—as a parent of secondary growths, the most antiquated. We find recorded in the pages of Holy Writ:—"Darkness was upon the face of the *deep*," and "God moved on the face of the *waters*." (Gen. i., 2.) It was not until the waters were formed that the firmament (heavens) was made. The waters existed before even dry land appeared. When that grand scheme of nature had been fulfilled—when all the plants of the earth, and lights, had been created, to balance the laws of the sphere—there was a stillness, such as never can be equaled again.

Then, on the fifth day, "God said, let the *waters* bring forth abundantly the moving creatures that hath life;" not only "whales and every living thing which the waters brought forth after his kind," but "the fowl that may fly above the earth in the open firmament of heaven."

